# Kinematic variability, fractal dynamics and local dynamic stability of treadmill walking

**DOI:** 10.1186/1743-0003-8-12

**Published:** 2011-02-24

**Authors:** Philippe Terrier, Olivier Dériaz

**Affiliations:** 1 IRR, Institut de Recherche en Réadaptation, Sion, Switzerland; 2 Clinique Romande de Réadaptation SuvaCare, Sion, Switzerland

## Abstract

**Background:**

Motorized treadmills are widely used in research or in clinical therapy. Small kinematics, kinetics and energetics changes induced by Treadmill Walking (TW) as compared to Overground Walking (OW) have been reported in literature. The purpose of the present study was to characterize the differences between OW and TW in terms of stride-to-stride variability. Classical (Standard Deviation, SD) and non-linear (fractal dynamics, local dynamic stability) methods were used. In addition, the correlations between the different variability indexes were analyzed.

**Methods:**

Twenty healthy subjects performed 10 min TW and OW in a random sequence. A triaxial accelerometer recorded trunk accelerations. Kinematic variability was computed as the average SD (MeanSD) of acceleration patterns among standardized strides. Fractal dynamics (scaling exponent α) was assessed by Detrended Fluctuation Analysis (DFA) of stride intervals. Short-term and long-term dynamic stability were estimated by computing the maximal Lyapunov exponents of acceleration signals.

**Results:**

TW did not modify kinematic gait variability as compared to OW (multivariate T^2^, p = 0.87). Conversely, TW significantly modified fractal dynamics (t-test, p = 0.01), and both short and long term local dynamic stability (T^2 ^p = 0.0002). No relationship was observed between variability indexes with the exception of significant negative correlation between MeanSD and dynamic stability in TW (3 × 6 canonical correlation, r = 0.94).

**Conclusions:**

Treadmill induced a less correlated pattern in the stride intervals and increased gait stability, but did not modify kinematic variability in healthy subjects. This could be due to changes in perceptual information induced by treadmill walking that would affect locomotor control of the gait and hence specifically alter non-linear dependencies among consecutive strides. Consequently, the type of walking (i.e. treadmill or overground) is important to consider in each protocol design.

## Introduction

Walking is a repetitive movement which is characterized by a low variability [1]. This motor skill requires not only conscious neuromotor tasks but also complex automated regulation, both interacting to produce steady gait pattern. Classically, gait variability (i.a. kinematic variability) has been assessed from the differences among the strides (Standard Deviation SD, coefficient of variation CV), i.e. each stride considered as an independent event resulting from a random process. However, this approach fails to account for the presence of feedback loops in the motor control of walking: the walking pattern at a given gait cycle may have consequences on subsequent strides. As a result, correlations between consecutive gait cycles and non-linear dependencies are expected.

During the last decades, various new mathematical tools have been used to better characterise the non-linear features of gait variability. With the Detrended Fluctuation Analysis (DFA [2-4]) it has been observed that the stride interval (i.e. time to complete a gait cycle) at any time was related (in a statistical sense) to intervals at relatively remote times (persistent pattern over more than 100 strides). This dependence (memory effect) decayed in a power-law fashion, similar to scale-free, fractal-like phenomena (fractal dynamics [1,3-5]), also known as 1/f^β ^noise [6]).

Another non-linear approach was proposed to characterize the dynamic variability in continuous walking. The sensitivity of a dynamical system to small perturbations can be quantified by the system maximal Lyapunov exponent, which characterizes the average rate of divergence in pseudo-periodic processes [7]. This method allows to evaluate the ability of locomotor system to maintain continuous motion by accommodating infinitesimally small perturbations that occur naturally during walking [8]. This includes external perturbations induced by small variations in the walking surface, as well as internal perturbations resulting from the natural noise present in the neuromuscular system [8].

Many theoretical questions are still open about the validity and application of these methods. For instance, DFA results are difficult to interpret [9], and no definitive conclusion on the presence of long range correlations should be drawn relying only on it. In addition, the underlying mechanism of long range correlations in stride interval is not fully understood [3,10]. West & Latka suggested that the observed scaling in inter-stride interval data may not be due to long-term memory alone, but may, in fact, be due partly to the statistics [11]. It was also suggested that the use of multi-fractal spectrum could be a better approach than mono-fractal analysis, such as DFA [12,13]. There are also several methodological issues to compute consistent and reliable stability index [14,15].

In parallel with the ongoing theoretical research on non-linear analysis of physiological time series, the use of non-linear bio-markers in applied clinical research has been already fruitful. In the field of human locomotion, it has been demonstrated that gait variability could serve as a sensitive and clinically relevant tool in the evaluation of mobility and the response to therapeutic interventions. For instance, gait variability (SD and dynamics) is altered in clinically relevant syndromes, such as falling and neuro-degenerative disease [16,17]. Gait instability measurement apparently predict falls in idiopathic elderly fallers [18]. Improvements in muscle function are associated with enhanced gait stability in elderly [19].

Motorized treadmills are widely used in biomechanical studies of human locomotion. They allow the documentation of a large number of successive strides under controlled environment, with a selectable steady-state locomotion speed. In the rehabilitation field, treadmill walking is used in locomotor therapy, for instance with partial body weight support in spinal cord injury or stroke rehabilitation [20,21]. Since the classical work of Van Ingen Schenau [22], it is admitted that overground and treadmill locomotion are similar if treadmill belt speed is constant. Nevertheless, both walking types present small differences in kinematics [23,24], kinetics [25] and energetics [26]. It was also observed that treadmill locomotion induced shorter step lengths and higher cadences than walking on the floor at the same speed [26,27]. There is still a matter of debate to interpret such subtle differences [28,29].

It is obvious that treadmill walking (TW) induces specific kinaesthetic and perceptual information. Previous studies confirmed that vision plays a central role in the control of locomotion [30,31]. These differences in visual afferences between TW and Overground Walking (OW) may induce a modification in motor control, and consequently in gait variability.

In 2000, Dingwell et al. analyzed TW local dynamic stability (maximal Lyapunov exponent) in 10 healthy subjects [8,32]. They highlighted significant differences between TW and OW by evaluating local dynamic stability of lower limbs kinematics [8]. The effect was low in upper body accelerations. Later [32], they calculated more specifically short term stability and found a strong effect of TW in trunk accelerations. On the other hand, they found a greater kinematic variability at the lower limb level in OW as compared to TW, but no significant difference in trunk kinematics.

In 2005, Terrier et al. [1], by using high accuracy GPS, described low stride-to-stride variability of speed, step length and step duration in free walking. They observed that the constraint of rhythmical auditory signal ("metronome walking") did not alter kinematic variability, but modify the fractal dynamics (DFA) of the stride interval (anti-persistent pattern).

Based on these previous works, the working hypothesis of the present article is 1) that the constraint of TW (constant speed, narrow pathway) may induce a less persistent pattern in the stride interval, by analogy to the constraint induced by a metronome; 2) that TW may increase the local dynamic stability of walking, due to the diminution of degrees of freedom in the more constrained artificial environment [32,33], 3) that, for the same reasons, TW may slightly reduce kinematic variability [32,33] 4) that no correlation exist between the 3 variability indexes, because they are related to different aspects of the locomotion process.

The purpose of the present study was to analyze, by using trunk accelerometry, differences between TW and OW in terms of stride-to-stride kinematic variability (SD), fractal dynamics (by DFA) and local dynamic stability (maximal Lyapunov exponent). In addition, we assessed the strength of the relationships between these variables (canonical correlation analysis).

## Methods

### Participants

Twenty healthy male subjects, with no neurological deficit or orthopaedic impairment, participated to the study. Most of them were recruited among participants of a previous "treadmill" study implying only males subjects [34]. Their characteristics were (mean ± SD): age 35 ± 7 yr, body mass 79 ± 10 kg, and height 1.80 ± 0.06 m. All subjects were well trained to walk on a treadmill before the beginning of the study. The experimental protocol was approved by the local ethics committee (commission d'éthique du Valais).

### Apparatus

The motion sensor (Physilog system, BioAGM, Switzerland [35]) was a triaxial accelerometer connected to a data logger recording body accelerations in medio-lateral (ML), vertical (V) and antero-posterior (AP) directions. The dimensions of the logger were 130 × 68 × 30 mm and the weight was 285 g. The accelerometers are piezoresistive sensors coupled with amplifiers (± 5 g, 500 mV/g) and mounted on a belt. The signals were sampled at 200 Hz with 12-bit resolution. After each experiment, the data were downloaded to a PC computer and converted in earth acceleration units (g) according to a previous calibration. Data analysis was then performed by using Matlab (Mathworks, Natick MA, USA) and Stata 11.0 (StataCorp LP, TX, USA)

### Procedures

The subjects performed 10 min. treadmill walking (TW) and 10 min. overground walking (OW) in a random order. A rest period of five minutes (sitting still) was imposed between the two trials. The motor-driven treadmill was a Technogym, (Runrace, Italy). The imposed speed was 1.25 m/s (4.5 km/h) for all subjects: in the context of a previous study [34], we assessed average running and walking preferred speed on the same treadmill in 88 male subjects; an average of 1.26 ± 0.13 m/s was observed. A thirty second warm-up was performed before the beginning of the measurement. For the OW test, the subjects walked along a standardized 800 m indoor circuit along hospital corridors and halls. The circuit exhibited only 90° turns. A large part (about 400 m) of the circuit was constituted by a long corridor. Other people working in the hospital were present in the halls. Hence, the OW trials mimicked actual condition of walking. Subects were asked to walk at their Preferred Walking Speed (PWS) with a regular pace. Under both conditions, the accelerometer was attached to the low back (L4-L5 region) with an elastic belt, and the logger was worn on the side of the body. Subjects wore their own low-rise comfortable walking shoes.

### Stride intervals and kinematic variability

**Figure 1:**
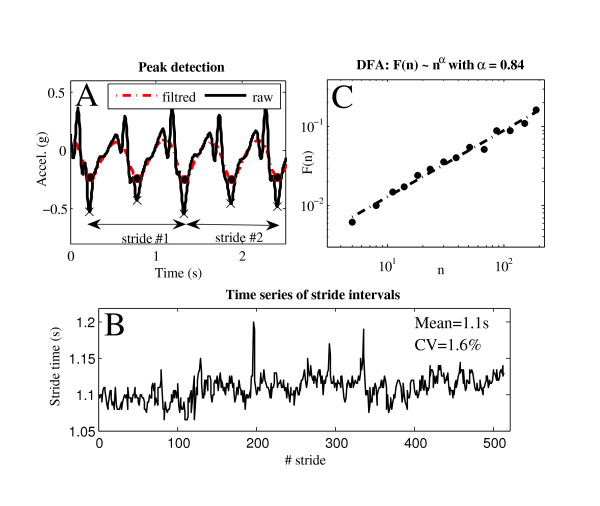
**Method: Step detection, stride intervals and Detrended Fluctuation Analysis**. One subject performed 10 min of free walking. **A: **2.5s sample of the antero-posterior acceleration signal; red dotted line is a low pass filtered (<3 Hz) version of the raw signal (black continuous line). Cross and black circle indicate how the algorithm specifically detect the heel strike (see method section for further explanation). The duration of two consecutive steps is defined as stride interval. **B: **Time series of stride intervals during the 10 min walking test. Average stride time (mean) and CV (SD/mean * 100) is also presented. **C: **Detredend Fluctuation Analysis (DFA). The fractal dynamics of the time series (B) is characterized by the scaling exponent α, computed by comparing the fluctuation (F(n)) at different scales (n) in a log-log plot.

Five seconds were removed at the beginning and at the end of the 10 min. acceleration measurements in order to avoid non-stationary periods. Heel strike was detected in the raw acceleration AP signal with a peak detection method designed to minimize the risk of false step detection: first, we generated a low-pass filtered version of the signal (4 order Butterworth, 3 Hz, zero-phase filtering). The time of each local minimum was detected. By superimposing the Filtered Signal (FS) to the original, Unfiltered Signal (US), we tracked the nearest peak in US of each local minimum in FS. US peak time was then chosen as the limit between two steps (Figure 1A). The strides were defined as two consecutive steps. On average, the number of strides was 543 per trial.

**Figure 2:**
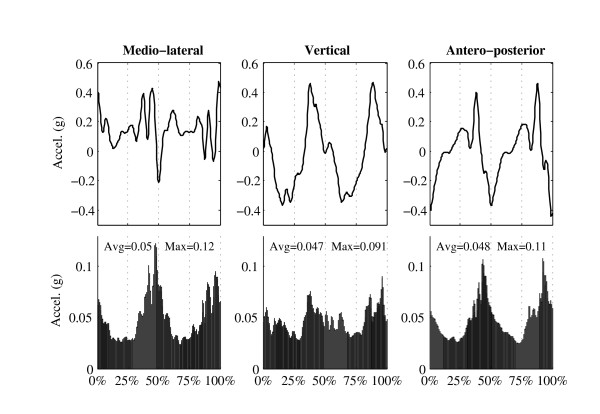
**Method: variability, MeanSD**. One subject (same as in Figure 1) performed 10 min of free walking. Each stride (see Figure 1A) was normalized to 200 samples (0% to 100% gait cycle). **Top: **Average acceleration pattern of the normalized strides (N = 513). **Bottom: **Standard Deviation (SD) of the normalized strides (N = 513). MeanSD is the average SD of the 200 samples.

Time series of the stride intervals were used to compute a traditional variability index (Coefficient of Variation of the stride time, CV = SD/Mean*100, Figure 1B). Moreover, the variability of the acceleration pattern among strides was evaluated as follows (Figure 2): each stride was normalized to 200 sample points by using a polyphase filter implementation (Matlab command *Resample*); the average stride-to-stride Standard Deviation across all data points ((SD(i) ∀ i ∈ [1 ....200])) was evaluated (MeanSD = 〈SD(i)〉).

### Detrended Fluctuation Analysis

The presence of long range correlations in the time series of stride intervals (fractal dynamics) was assessed by the use of the non-linear DFA method. Strictly speaking, this non-linear method should be used in addition to other statistical tools to definitively conclude that a process is a true 1/f^β ^noise with power-law decrease of long range auto-correlations [6,9]. However, DFA has been successfully used as relevant biomarker in numerous studies [1,16,17,36,37]. Detrended Fluctuation Analysis is based on a classic root-mean square analysis of a random walk, but is specifically designed to be less likely affected by nonstationarities. Full details of the methodology are published elsewhere [1-4]. In short, the integrated time series of length *N *is divided into boxes of equal length, *n*. In each box of length *n*, a least squares line is fit to the data (representing the trend in that box). The y coordinate of the straight line segments is denoted by y_*n*_(*k*). Next, the integrated time series, y(*k*), was detrended, by subtracting the local trend, y_*n*_(*k*), in each box. The root-mean-square fluctuation of this integrated and detrended time series is calculated by

(1) $$F(n)=\sqrt{\frac{1}{N}\sum_{k=1}^{N}{\left[y(k)-y_{n}(k)\right]}^{2}}$$

This computation is repeated over all box sizes (from 4 to 200) to characterize the relationship between F(*n*), the average fluctuation, and the box size, *n*. The fluctuations can be characterized by the scaling exponent *α*, which is the slope of the line relating log F(*n*) to log(*n*) (*F(n) ~ n*^*α*^), Figure 1C). Long range correlations are present in the original time series when *α *lies between 0.5 and 1 [3,4].

In a finite length time series, an uncorrelated process could exhibit "by chance" a scaling exponent different from the theoretical 0.5 value. To statistically differentiate the stride time series from a random uncorrelated process, we applied the surrogate data method [1,3]. This method increases the confidence that the analyzed series exhibits long-range correlation. Twenty different surrogate data sets were generated by shuffling the original time series in a random order. On each data set, DFA analysis was performed to calculate *α *value. The standard deviation and mean of this sample was calculated and compared to *α *exponent of the original series. The result is considered significant if the original *α *is 2 standard deviation away from the mean of the surrogate data set.

### Local dynamic stability

**Figure 3:**
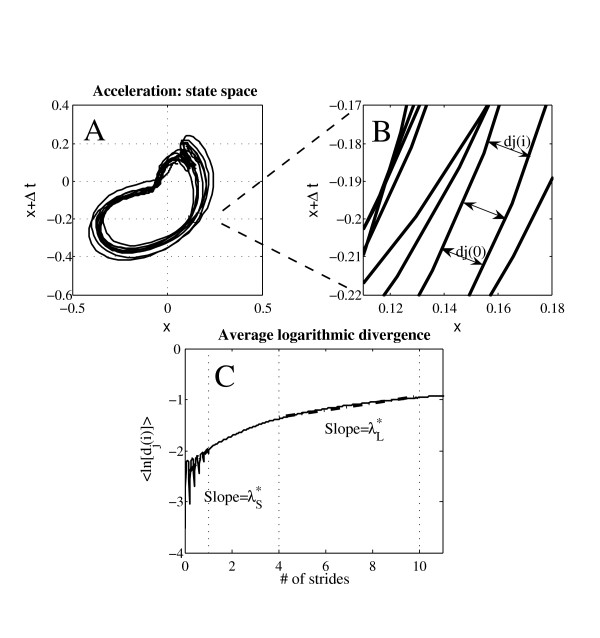
**Method: dynamic stability, maximal Lyapunov exponent**. **A: **Two dimensional state space of the antero-posterior acceleration signal (5s) reconstructed from the original data set and its time delayed copy (Δt = 11 samples). **B: **Magnification of the state space. An initial local perturbation at dj(0) diverge across i time steps as measured by dj(i). **C: **Short term (λ_S_*) and long term (λ_L_*) finite-time maximal Lyapunov exponent computed from average logarithmic divergence.

The method for quantifying the local dynamical stability of the gait by using largest Lyapunov exponent has been extensively described in literature [8]. It examines structural characteristics of a time series that is embedded in an appropriately constructed state space. A valid state space contains a sufficient number of independent coordinates to define the state of the system unequivocally [38]. According to the Takens' theorem, an appropriate state space can be reconstructed from a single time series using the original data and its time delayed copies (figure 3A) [38].

(2) $$X(t)=[x(t),x(t+T),x(t+2T),\ldots ,x(t+(d_{E}-1)T]$$

Where *X(t) *is the *d*_*E*_-dimensional state vector, *x(t) *are the original data, *T *is the time delay, and *d*_*E *_is the embedding dimension. The time delays (*T*) were calculated individually for each of the 120 acceleration data set (3-axis, 2 conditions, and 20 individuals) from the first minimum of the Average Mutual Information (AMI) function [8,39]. Embedding dimensions (*d*_*E*_) were computed from a Global False Nearest Neighbors (GFNN) analysis [8,40]. Because the result was similar for all acceleration time series, we use a constant dimension (*d*_*E *_= 6) [8,32]]. The Lyapunov exponent is the mean exponential rate of divergence of initially nearby points in the reconstructed space (Figure 3B). Because the determination of the maximal Lypunov exponent requires intensive computing power, 7 min of the 10 min walking test (from 1.5 to 8.5 min.) was selected and the raw data were down-sampled to 100 Hz. The determination of the Lyapunov exponent was then achieved by using the algorithm introduced by Rosenstein and colleagues [7], which provided dedicated software to compute divergence as a function of time in finite-time series [41] (Figure 3B). The maximum finite-time Lyapunov exponents (λ*) were estimated from the slopes of linear fits in the divergence diagrams (Figure 3C). Strictly speaking, because divergence diagrams (Figure 3C) are non-linear, multiple slopes could be defined and so no true single maximum Lyapunov exponent exists. The slopes (exponents) quantify local divergence (and hence stability) of the observed dynamics at different time scale, and should not be interpreted as a classical maximal Lyapunov exponent in chaos theory.

Since each subject exhibited a different average step frequency, the time was normalized by average stride time for each subject and each condition (Figure 3C). As suggested by Dingwell and colleagues [32], we use two different time scales for assessing short-term and long-term dynamic stability: short term exponents (λ_S_*) was computed over the first stride (0 to 1), and long term exponents (λ_L_*) between 4 and 10 strides (Figure 3C).

### Statistical analysis

Mean and Standard Deviation (SD) were computed to describe the data (table 1). Ninety-five percent Confidence Intervals (CI) were calculated as ± 1.96 times the Standard Error of the Mean (SEM, N = 20).

**Figure 4:**
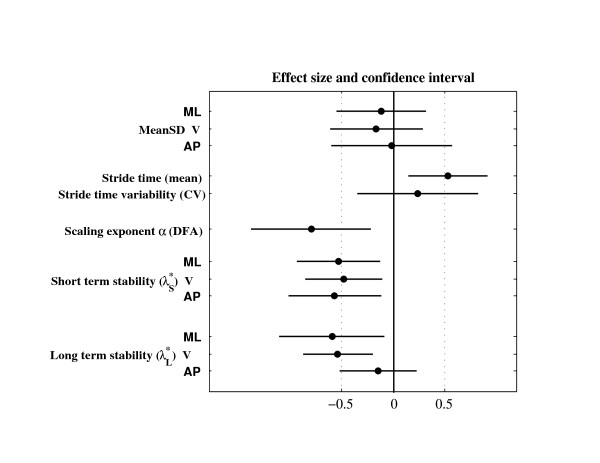
**Differences between overground and treadmill walking**. Effect size and confidence intervals. Black circles are the standardized effect size (Hedge's g), as reported in table 1. Horizontal lines are the 95% confidence intervals. The arbitrary limit of 0.5 (vertical dotted line) corresponds to a medium effect as defined by Cohen.

**Figure 5:**
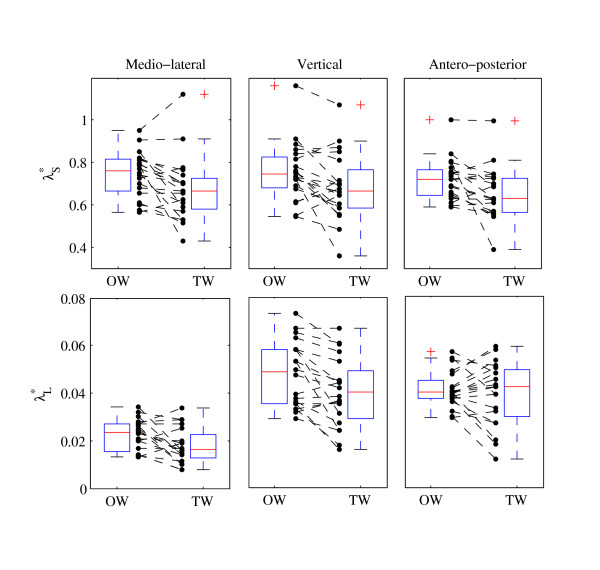
Individual changes of dynamic stability (λ*). Lyapunov exponent λ_L_* and λ_S_* of the 20 subjects are presented for Overground Walking (OW) and Treadmill Walking (TW). Discontinuous lines join OW and TW results. Boxplots show the quartiles and the median.

The effect size of TW as compared to OW was expressed in both absolute (mean difference) and standardized (mean difference divided by SD) terms. The standardized effect size was the Hedge's g, which is a modified version of the Cohen's d for inferential measure [42]. Paired t-tests between OW and TW were performed, and the p-values are shown in the last column of table 1. The precision of the effect sizes was estimated with CI (Figure 4). CI were ± 1.96 times the asymptotic estimates of the standard error (SE) of g [42]. The arbitrary limit of 0.5 was uses to delineate small effect size, as defined by Cohen [42]. The extent of the data (quartiles and median) and individual differences between conditions are shown in Figure 5 for λ*. In order to facilitate results interpretation by reducing the risk of type I statistical error, a Hotelling T^2 ^test was used. This is a multivariate generalization of paired t-test [43]. The null hypothesis is that a vector of p differences is equal to a vector of zeros. Two multivariate sets were tested: meanSD (p = 3) and λ* (p = 6).

Canonical correlation analyses (CCA, table 2&3) were performed in order to assess the strength of the relationships between different sets of variables [43]. This multivariate method allows one to find linear combinations (variates) in two sets of variables, which have maximum correlation (canonical correlation coefficient or canonical root) with each other. For each condition (OW and TW), two sets of p variables were analyzed: kinematic variability (set#1, p = 3) including MeanSD in ML, V and AP directions, and dynamic stability (set#2, p = 6), including short term and long term lyapunov exponent (λ_S_*, λ_L_*) in ML, V and AP directions. In addition, *α *scaling exponent was also analyzed with the same method vs. set#1 and set#2. In this case, CCA is equivalent to multiple regression analysis. Significance of the canonical correlations was assessed with the Wilks' lambda statistics.

To enhance the interpretation of CCA, different parameters were computed: the standardized canonical weights are the linear coefficients for each set after Z-transform of the variables; canonical loadings are the correlation coefficients between each variable and their respective linear composites; redundancy expresses the amount of variance in one set explained by a linear composite of the other set.

## Results

### Treadmill effect

As presented in table 1, TW did not modify the stride-to-stride kinematic variability of normalized acceleration pattern, either considering multivariate T^2 ^statistics (p = 0.87) or individual results for each direction. TW was on average performed at slightly lower cadence than Overground Walking (OW, 3% relative difference). The variability of stride interval was similar under both conditions. DFA of stride intervals revealed that TW changed the fractal dynamics of walking (-11% relative difference). Globally, multivariate analysis showed that the data are compatible with the assumption that TW modified dynamic stability of the gait (T^2 ^(6, 20) p = 0.0002). Five from six particular λ* exponents exhibited significant differences.

**Table 1:** Comparison between Overground and Treadmill Walking

		Overground Walking		Treadmill Walking		Effect Size		T-test	**T**^**2**^**-test**
N = 20		Mean ± SD	Confidence interval	Mean ± SD	Confidence interval	**Abs**.	**Norm**.	p	p
	ML	0.08 ± 0.03	0.07 - 0.09	0.07 ± 0.03	0.06 - 0.09	0.00	-0.12	0.59	
Mean variability (SD, g)	V	0.08 ± 0.03	0.07 - 0.09	0.08 ± 0.03	0.06 - 0.09	-0.01	-0.16	0.48	0.87
	AP	0.08 ± 0.03	0.07 - 0.09	0.08 ± 0.03	0.07 - 0.09	0.00	-0.01	0.96	

Stride time (mean, s)		1.06 ± 0.06	1.04 - 1.09	1.10 ± 0.07	1.07 - 1.13	0.03	0.53	**0.01**	
Stride time variability (CV, %)		2.74 ± 0.87	2.36 - 3.12	3.03 ± 1.44	2.40 - 3.66	0.29	0.24	0.43	
Scaling exponent α (DFA)		0.81 ± 0.09	0.78 - 0.85	0.72 ± 0.13	0.67 - 0.78	-0.09	-0.80	**0.01**	

	ML	0.75 ± 0.11	0.70 - 0.79	0.68 ± 0.15	0.61 - 0.74	-0.07	-0.53	**0.01**	
Short term stability (λ*_S_)	V	0.75 ± 0.14	0.69 - 0.82	0.68 ± 0.16	0.61 - 0.75	-0.07	-0.48	**0.01**	
	AP	0.72 ± 0.10	0.68 - 0.76	0.66 ± 0.13	0.60 - 0.71	-0.06	-0.57	**0.02**	**0.00**
	
	ML	0.022 ± 0.007	0.019 - 0.025	0.018 ± 0.008	0.015 - 0.021	-0.004	-0.60	**0.02**	
Long term stability (λ*_L_)	V	0.048 ± 0.014	0.042 - 0.054	0.040 ± 0.015	0.034 - 0.046	-0.008	-0.54	**0.00**	
	AP	0.041 ± 0.008	0.038 - 0.044	0.039 ± 0.013	0.033 - 0.045	-0.002	-0.15	0.48	

The Descriptive statistics of variability indexes are expressed as mean, Standard Deviation (SD) and 95% Confidence Interval (mean ± 1.96 times the Standard Error of the Mean). The effect size is given as Absolute (Abs.) and Normalized (Norm.) values, i.e. respectively the difference between Overground (OW) and Treadmill (TW) conditions (Abs.) and the difference normalized by SD (Hedge's g). The t-test column shows the p values of paired t-tests between TW and OW conditions. T^2^-test is the Hotelling multivariate test by regrouping MeanSD and λ*. Significant results (p < 0.05) are printed in bold. ML, V and AP stand for respectively Medio-Lateral, Vertical and Antero-posterior, i.e. the 3 directions of the triaxial accelerometer.

Figure 4 shows the accuracy of the effect size estimation. Non-linear estimators of gait variability (α, λ*) exhibit mostly medium effect size.

Figure 5 shows the individual results of the local dynamic stability (λ*). Stability was clearly increased (lower λ*) for a majority of subjects except for long-range Antero-Posterior stability λ_L_*.

**Figure 6:**
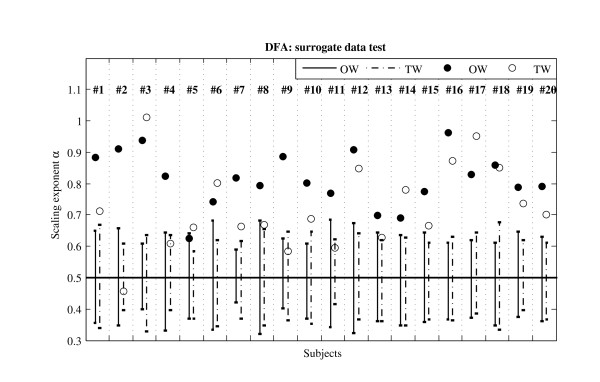
**Detrended Fluctuation Analysis: surrogate data tests**. The time series of stride intervals (Figure 1B) of each subject (#1 to #20) were analyzed by DFA (figure 1C) to determine the scaling exponent α indicating the presence of a long range correlation pattern in stride intervals. Black and white circles are respectively the scaling exponent for Overground Walking (OW) and Treadmill Walking (TW). Each time series was randomly shuffled twenty times to produce 20 surrogate time series. The average of these series is near 0.5 (random process with no correlation). The vertical bars show the extent of 2 times the SD of the 20 surrogate time series. Scaling exponent larger than this value can be considered significantly different from a random uncorrelated series.

Figure 6 presents the individual results of surrogate testing of fractal dynamics. The response to TW was not homogenous among subjects. Four subjects (20%) exhibited a significant turn of long range correlations to uncorrelated pattern. For ten more subjects (50%), a reduction was observed (more than 0.05), but outside the significant limits.

### Correlations

Table 2 shows the correlation matrix (Perason's r) of the variables under both conditions. It can be observed that correlations exist between the same variables measured along different axes (for instance MeanSD ML vs. MeanSD V, r = 0.92), what makes difficult the global interpretation of potential correlation among the different variability indexes.

**Table 2:** Correlation matrix

Overground Walking										Treadmill Walking									
**Correlation coefficients**:										**Correlation coefficients**:									
	**SD ML**	**SD V**	**SD AP**	**λ**_s_* ML	**λ**_s_* V	**λ**_s_* AP	**λ**_L_* ML	**λ**_L_* V	**λ**_L_* AP		**SD ML**	**SD V**	**SD AP**	**λ**_s_* ML	**λ**_s_* V	**λ**_s_* AP	**λ**_L_* ML	**λ**_L_* V	**λ**_L_* AP

**SD ML**	1.00									**SD ML**	1.00								
**SD V**	**0.95**	1.00								**SD V**	**0.92**	1.00							
**SD AP**	0.14	0.24	1.00							**SD AP**	**0.59**	**0.62**	1.00						
**λ**_s_* ML	0.05	-0.04	-0.02	1.00						**λ**_s_* ML	0.26	0.20	-0.26	1.00					
**λ**_s_* V	-0.20	-0.17	-0.04	**0.47**	1.00					**λ**_s_* V	-0.17	-0.08	**-0.50**	**0.74**	1.00				
**λ**_s_* AP	-0.27	-0.24	-0.37	**0.57**	**0.45**	1.00				**λ**_s_* AP	-0.01	0.08	-0.32	**0.77**	**0.75**	1.00			
**λ**_L_* ML	0.16	0.17	0.07	-0.13	0.06	-0.27	1.00			**λ**_L_* ML	**-0.51**	**-0.50**	**-0.56**	0.34	0.41	0.30	1.00		
**λ**_L_* V	-0.31	-0.37	0.10	-0.23	-0.24	-0.28	0.41	1.00		**λ**_L_* V	**-0.76**	**-0.81**	**-0.47**	-0.36	-0.14	-0.30	**0.53**	1.00	
**λ**_L_* AP	**-0.47**	**-0.52**	0.17	-0.13	-0.01	-0.36	**0.55**	**0.75**	1.00	**λ**_L_* AP	**-0.77**	**-0.82**	**-0.57**	-0.37	-0.18	-0.21	**0.45**	**0.86**	1.00
**α (DFA)**	**-0.45**	-0.35	-0.39	0.09	0.10	**0.51**	-0.09	0.08	-0.09	**α (DFA)**	**-0.56**	**-0.56**	-0.36	0.12	0.25	0.10	0.28	0.37	0.42

Pearson's r correlation coefficients between the variables. SD = Mean Standard Deviation (MeanSD). λ_S_* = maximal Lyapunov exponent, short term dynamic stability. λ_L_* = maximal Lyapunov exponent, long term dynamic stability. α = scaling exponent (Detrended Fluctuation Analysis), fractal dynamics. ML, V and AP stand for respectively Medio-Lateral, Vertical and Antero-posterior. Significant correlation are bold printed (p < 0.05).

In table 3, the results of 6 CCA are shown in details in order to explore global correlation hypotheses. The data seem compatible with the hypothesis that a negative correlation exists between kinematic variability (MeanSD) and local dynamic stability (λ*) under TW condition. Namely, two significant canonical roots (R^2 ^= 0.88 and 0.62) indicates that the canonical variates share an important variance. In addition, the canonical loadings show that the canonical model extract a substantial portion of the variance from the variables (70% from the set#1 and 27% from the set#2). Finally, the redundancy analysis reveals that at least 70% of the variance of the set#2 (stability) can be explained by the set#1 (kinematic variability). The five other CCA did not produce clear evidence for significant relationship between the analyzed sets of variables. Three CCA showed low and non significant canonical roots. Two CCA exhibited barely significant correlation, but the analysis of loadings showed that the canonical model did not explain a large part of the variances in the sets.

**Table 3:** Canonical Correlation Analysis (CCA)

**Overground Walking**								**Treadmill Walking**							
**Standardized weights**				**Loadings**				**Standardized weights**				**Loadings**			
			
Set #1	1	2	3	Set #1	1	2	3	Set #1	1	2	3	Set #1	1	2	3
			
**SD ML**	-0.06	2.76	1.70	**SD ML**	-0.95	0.30	0.07	**SD ML**	-1.30	2.29	0.04	**SD ML**	-0.94	-0.10	0.33
**SD V**	-0.98	-2.72	-1.62	**SD V**	-0.98	0.09	-0.18	**SD V**	0.63	-2.39	1.07	**SD V**	-0.80	-0.46	0.38
**SD AP**	0.20	0.79	-0.70	**SD AP**	-0.04	0.52	-0.85	**SD AP**	-0.38	-0.31	-1.18	**SD AP**	-0.76	-0.42	-0.49
			
Set #2	1	2	3	Set #2	1	2	3	Set #2	1	2	3	Set #2	1	2	3
			
**λ**_s_* ML	-0.26	1.02	0.65	**λ**_s_* ML	0.04	0.34	0.65	**λ**_s_* ML	-0.78	1.77	0.42	**λ**_s_* ML	-0.13	0.27	0.85
**λ**_s_* V	0.12	-0.07	-0.68	**λ**_s_* V	0.19	-0.16	-0.14	**λ**_s_* V	0.79	-0.39	0.63	**λ**_s_* V	0.39	-0.08	0.81
**λ**_s_* AP	0.52	-1.04	0.66	**λ**_s_* AP	0.20	-0.53	0.69	**λ**_s_* AP	0.25	-0.79	-0.23	**λ**_s_* AP	0.20	-0.15	0.74
**λ**_L_* ML	-0.73	-0.22	0.11	**λ**_L_* ML	-0.18	0.05	-0.19	**λ**_L_* ML	0.20	-0.52	0.06	**λ**_L_* ML	0.59	0.24	0.17
**λ**_L_* V	-0.05	0.34	0.28	**λ**_L_* V	0.45	0.31	-0.01	**λ**_L_* V	-0.01	0.39	-0.99	**λ**_L_* V	0.69	0.42	-0.55
**λ**_L_* AP	1.23	-0.02	-0.21	**λ**_L_* AP	0.63	0.35	-0.25	**λ**_L_* AP	0.57	0.77	0.67	**λ**_L_* AP	0.75	0.45	-0.38
			
Can. correlations				Redundancy				Can. correlations				Redundancy			
			
	**0.89**	0.73	0.28	Set #1	0.50	0.07	0.02		**0.94**	**0.79**	0.62	Set #1	0.62	0.08	0.06
p	0.01	0.30	0.89	Set #2	0.09	0.06	0.01	p	0.00	0.03	0.15	Set #2	0.24	0.06	0.15
			
Standardized weights				Loadings				Standardized weights				Loadings			
											
	1				1				1				1		
											
**α (DFA)**	1.00			**α (DFA)**	1.00			**α (DFA)**	1.00			**α (DFA)**	1.00		
											
Set #2	1			Set #2	1			Set #2	1			Set #2	1		
											
**λ**_s_* ML	-0.40			**λ**_s_* ML	0.15			**λ**_s_* ML	0.64			**λ**_s_* ML	0.21		
**λ**_s_* V	-0.10			**λ**_s_* V	0.16			**λ**_s_* V	0.65			**λ**_s_* V	0.43		
**λ**_s_* AP	1.20			**λ**_s_* AP	0.84			**λ**_s_* AP	-0.39			**λ**_s_* AP	0.18		
**λ**_L_* ML	0.01			**λ**_L_* ML	-0.14			**λ**_L_* ML	-0.46			**λ**_L_* ML	0.49		
**λ**_L_* V	0.42			**λ**_L_* V	0.13			**λ**_L_* V	0.22			**λ**_L_* V	0.65		
**λ**_L_* AP	-0.09			**λ**_L_* AP	-0.15			**λ**_L_* AP	1.01			**λ**_L_* AP	0.73		
											
Can. correlations				Redundancy				Can. correlations				Redundancy			
											
	0.61			α (DFA)	0.38				0.58			α (DFA)	0.34		
p	0.32			Set #2	0.05			p	0.42			Set #2	0.08		
											
Standardized weights				Loadings				Standardized weights				Loadings			
											
	1				1				1				1		
											
**α (DFA)**	1.00			**α (DFA)**	1.00			**α (DFA)**	1.00			**α (DFA)**	1.00		
											
Set #1	1			Set #1	1			Set #1	1			Set #1	1		
											
**SD ML**	-2.32			**SD ML**	-0.67			**SD ML**	-0.51			**SD ML**	-0.98		
**SD V**	1.85			**SD V**	-0.52			**SD V**	-0.50			**SD V**	-0.98		
**SD AP**	-0.70			**SD AP**	-0.58			**SD AP**	-0.02			**SD AP**	-0.63		
											
Can. correlations				Redundancy				Can. correlations				Redundancy			
											
	**0.67**			α (DFA)	0.45				0.58			α (DFA)	0.33		
p	0.02			Set #1	0.16			p	0.09			Set #1	0.26		

Canonical correlation analysis between 6 sets of variables. SD = Mean Standard Deviation. λ_S_* = maximal Lyapunov exponent, short term dynamic stability. λ_:_* = maximal Lyapunov exponent, long term dynamic stability. α = scaling exponent (Detrended Fluctuation Analysis), fractal dynamics. ML, V and AP stand for respectively Medio-Lateral, Vertical and Antero-posterior.

## Discussion

The purpose of the present study was to analyze three gait variability indexes under two walking conditions in order to highlight modifications induced by motorized treadmill and to analyze the relationship between the indexes.

According to the working hypothesis, the results are summarized as follows:

1) As compared to Overground Walking (OW), Treadmill Walking (TW) significantly reduced the average scaling exponent (lower α), but did not reverse the correlated pattern to a random or anti-persistent pattern in a majority of subjects.

2) TW significantly increased local dynamic stability (lower λ*).

3) TW did not significantly modify the kinematic variability (MeanSD).

4) No evident relationship was observed between variability indexes during OW at preferred walking speed, but in TW significant negative correlation was found between kinematic variability (MeanSD) and stability (λ*).

Overall, Conventional variability analysis (MeanSD) failed to report differences between OW and TW, whereas non-linear approaches were able to show significant changes. The variability indexes were poorly correlated together (with one exception), which might signify that each index was related to a different aspect of motor control.

### Technical issues

For the present study, portable trunk accelerometry was chosen because it offers the possibility to record long-term free walking. Hence, the results concern the gait stability measured from accelerations of the low-back. Comparisons with other results should take into account that that the different gait stability studies use different kinematic variables (acceleration [7,10], positions [44], angle [8]) and different body location (thorax, head, knee, and ankle) to assess λ*. We found λ* similar to those measured by others [8,32], suggesting that the results are rather independent on the measurements methods.

In fractal dynamics studies, the first step is the detection of the periodic pattern of the gait in order to compute time series of stride intervals. Several methodologies have been used to measure long-term time series of stride intervals, such as foot switches [3,5], goniometer [45], video analysis [46], or high accuracy GPS [1]. Because the same variable is used (i.e. time duration of the gait cycle) for DFA analyses, data from different studies are probably comparable.

In order to increase the likelihood to point out significant correlations among variability indexes, we designed the experiment to obtain a substantial degree of standardization: we imposed the same speed (1.25 m/s, 4.5 km/h) for all subjects on the treadmill. This speed was chosen on the basis of a previous experiment (partially published yet [34]), which showed that the preferred speed in the same experimental conditions (same room, same treadmill) was 1.26 ± 0.13 m/s (n = 88). Similar values are found in the literature: 1.25 m/s (n = 8) [47], 1.19 m/s (n = 26) [48].

Walking speed was not standardized between TW and OW, as in other studies [32]. However, by selecting treadmill speed at the same speed of overground preferred speed, the results would be that subjects walk at higher speed than their preferred speed on the treadmill. Several studies showed a substantial difference between both conditions: Dal et al. [48] demonstrated that preferred walking speed determined on a treadmill is slower than overground (21% relative difference); Marsh et al. [49]showed that, when older adults were allowed to choose a preferred walking pace, they walked faster (+61%), used longer strides, and had a faster rate walking overground than when they walked on a treadmill. As a result, speed normalization could introduce unwanted bias. Our experimental design was therefore a compromise, which standardized speed among subjects in TW condition, but also which selected walking speed close to preferred speed, making both OW and TW conditions comparable.

In addition, Indirect clues seem to indicate that TW and OW conditions were quite similar: 1) stride time (which is related to walking speed) were close (3% difference, small effect size), 2) stride time variability (CV) was the same (no significant differences), 3) no correlation was observed between stride time and other parameters (results not shown),

### Differences between treadmill and overground walking

Kinematic variability, fractal dynamics (DFA) and local dynamic stability (Lyapunov exponents) quantify different aspects of locomotor control [32]. Kinematic variability describes the range in which the locomotor system operates. DFA quantify temporal dynamics of discrete events (i.e stride interval) over hundreds of consecutives strides; it assesses the presence of long-range correlations between strides, and hence analyzes the characteristics of feedbacks in locomotor control. Lyapunov exponents quantify the temporal dynamics in continuous time based on the theory of deterministic chaos; it evaluates the degree of divergence in the signal, and hence the resilience of the locomotor system to small perturbations. Therefore, it can be expected that these variability indexes did not react in the same way under various conditions.

These assumptions were experimentally verified in various studies that observed changes of λ* and kinematic variability between different experimental conditions or between different populations. For instance it was observed that patients with peripheral neuropathy present altered dynamic stability but normal kinematic variability [33,50]. Other investigators have shown that an exercise training intervention in elderly people could improve dynamic stability but not decrease kinematic variability [19].

Despite differences in the method of measurement (low-back vs. thorax acceleration) and in the experimental design (speed normalization), our results are generally in accordance with the results of Dingwell et al [32]. They analyzed only 10 healthy individuals, therefore statistical significance for small effects was more difficult to reach than in the present study. They showed a significant treadmill effect in short-term stability (lower λ_S_*). A slight but not significant effect for long-term vertical stability (lower λ_L_*) was found. They observed that kinematic variability (MeanSD) for upper body accelerations was generally greater for OW than TW, but this trend was only significant for antero-posterior accelerations. They explained that underlying causes of differences between TW and OW were unclear: on one hand, the motorized treadmill imposed a constant nominal speed on the subjects and constrained them to walk along a much narrower and straighter path than during OW; but on the other hand, differences may have been induced by intra-stride fluctuations in treadmill belt speed, differences in mechanical compliance between the walking surfaces, and changes in visual and vestibular perceptual information. In light of the results of the present study, we hypothesize that motor control is able to maintain the same range of kinematic variability in both TW and OW conditions (same kinematic variability), probably because of compensating effects: in TW, destabilizing factors (intra-stride belt speed fluctuations, disturbing mechanical compliance, alteration of perceptual information) are balanced by stabilizing factors (constant speed, narrow and straight path). Conversely, motor control strategy adapting the gait to TW seems to specifically alter non-linear dependencies among consecutive strides: the stabilizing factors override the destabilizing ones.

In a subsequent study, Dingwell & Marin [51] analyzed speed effect on dynamical stability (λ_S_* and λ_L_*) and kinematic variability (MeanSD). Walking speed was normalized by individual PWS on a treadmill. Speed range was 0.6PW to 1.4PWS by steps of 0.2. They found significant speed effect for both λ* and MeanSD: however the effect was small for 0.8-1.2 PWS. Under our experimental conditions [34], we observed that inter-indivudual variability of PWS on the treadmill was low: 90% of individuals walked in the range of 0.87-1.13 mean PWS. As a result, the speed effect among individuals in the present study was probably low. This is also indirectly confirmed by the low inter-individual variability of stride duration (CV = 6%).

Fractal dynamics of stride intervals has been extensively studied by Hausdorff et al. [36]. Them and other [1,3,52] have observed that constrained walking (paced cadence with a metronome), deeply modified the scaling exponent. By analogy, because treadmill also constraints the gait by imposing a constant speed, a similar effect could be expected. The results of the present study showed, in a majority of subjects, a lowering of scaling exponent to a less correlated pattern. The effect was not as strong as with paced walking [1]. The explanation could be that treadmill constrained walking speed, while metronome constrained walking pace; it could be hypothesized that the adaptation of locomotor control to external cues specifically modify correlation pattern of the constrained walking parameter, as suggested by the results of Terrier et al. [1], but this remains to be investigated.

### Correlations between variability indicators

While fractal dynamics, local dynamic stability and kinematic variability characterize different features of gait variability, it is not excluded that relationships exists between them.

Jordan et al. [46] recently analyzed fractal dynamics and stability in walking/running transition on treadmill. They observed a positive correlation between λ_L_* and α (r^2 ^= 0.65, N = 12). They also observed that scaling exponent is minimal close to PWS [53] and suggested that "reduced strength of long range correlations at preferred locomotion speeds is reflective of enhanced stability and adaptability at theses speeds". Our results, using CCA, did not confirm this suggestion. No evident correlation between scaling exponent and dynamic stability was found. Several differences in the measurement method (trunk accelerometry vs 3D video analysis) and in the experimental design (high speed vs. moderate speed) may explain this divergence.

Previous studies have analyzed the relationships between variability (meanSD) and local dynamic stability (λ_S_* and λ_L_*). Dingwell et al. pointed out "the general lack of correlation between the standard deviation and λ* exponents" [32]. In contrast, other investigators recently observed significant positive correlation between λ_S_* and MeanSD [54]. The results of the present study showed a counterintuitive negative correlation between λ* and MeanSD: during treadmill walking (but not in OW), higher kinematic variability seemed to be related to higher local stability (i.e. low λ*). As explained above, the use of different methodologies is a potential source of divergence between studies concerning dynamic stability. It is not excluded that a confounding factor, not measured yet, related to both MeanSD and λ* could indirectly explain this correlation. Further investigations are needed to better understand the relationship between these two variability indexes.

## Conclusions

Scaling exponent (α) and maximal Lypunov exponent (λ*) have been advocated as a relevant indicator of neuromuscular control of stability during human locomotion [8,32,36,55]. The results of the present study showed that treadmill modified fractal dynamics (α) and local dynamic stability (λ*) of the gait, but not kinematic variability (MeanSD). This should be kept in mind when using motorized treadmill either for fundamental research or in locomotor therapies.

Whereas both scaling exponent (α) and maximal Lypunov exponent (λ*) are sensitive enough to identify differences between OW and TW, they seem not correlated together. This suggests that both indexes deserve to be used in conjunction when analyzing long term gait variability, because they describe different locomotor characteristics.

## Competing interests

The authors declare that they have no competing interests.

## Authors' contributions

PT performed measurements and data analysis, and drafted the manuscript. OD participated in the design and coordination of the study and assisted with drafting the manuscript. All authors read and approved the final manuscript.
